# Health, environmental and distributional impacts of cycling uptake: The model underlying the Propensity to Cycle tool for England and Wales

**DOI:** 10.1016/j.jth.2021.101066

**Published:** 2021-09

**Authors:** James Woodcock, Rachel Aldred, Robin Lovelace, Tessa Strain, Anna Goodman

**Affiliations:** a University of Cambridge, UK; b University of Westminster, UK; c University of Leeds, UK; d LSHTM, UK

**Keywords:** Cycling, Appraisal, Health impact modelling, Carbon, Equity

## Abstract

**Introduction:**

The Propensity to Cycle Tool (PCT) is a widely used free, open source and publicly available tool for modelling cycling uptake and corresponding health and carbon impacts in England and Wales. In this paper we present the methods for our new individual-level modelling representing all commuters in England and Wales.

**Methods:**

Scenario commuter cycling potential in the PCT is modelled as a function of route distance and hilliness between home and work. Our new individual-level approach has allowed us to create an additional “Near Market” scenario where age, gender, ethnicity, car ownership and area level deprivation also affect an individual's likelihood of switching to cycling. For this and other scenarios, we calculate the carbon benefits of cycling uptake based on the trip distance and previous mode, while health benefits are additionally affected by hilliness and baseline average mortality risk. This allows the estimation of how health and carbon benefits differ by demographic group as well as by scenario.

**Results:**

While cycle commuting in England and Wales is demographically skewed towards men and white people, women and people from ethnic minorities have greater cycling potential based on route distance and hilliness. Benefits from cycling uptake are distributed differently again. For example, while increasing female cycling mode share is good for equity, each additional female cyclist generates a smaller average health and carbon benefit than a male cyclist. This is based on women's lower baseline mortality risk, shorter commute travel distances, and lower propensity to commute by car than men.

**Conclusion:**

We have demonstrated a new approach to modelling that allows for more sophisticated and nuanced assessment of cycling uptake and subsequent benefits, under different scenarios. Health and carbon are increasingly incorporated into appraisal of active travel schemes, valuing important outcomes. However, especially with better representation of demographic factors, this can act as a barrier to equity goals.

## Highlights

• A sophisticated model underlies the Propensity to Cycle Tool (PCT) for England and Wales.
• Cycling uptake-test and its health and carbon impacts are simulated under a range of scenarios.
• Microsimulation allows analysis of demographic variation in uptake and benefits.
• While women and ethnic minorities cycle less, they make shorter more ‘cyclable’ trips.
• However, active mode appraisal methods risk placing lower value on their cycling uptake.

## 1 Background

Recognising the wide-ranging benefits of active transport, many local and national governments have set cycling targets. England's national target (now ‘aim’) is to double the number of cycle journey stages by 2025, compared with 2013 (Department for Transport, 2017). Mode switch to cycling has multiple benefits. Starting cycling for everyday trips increases physical activity and hence reduces risk of non-communicable diseases.

Despite well-documented benefits, England's policy makers and planners have failed at the national level to get people cycling, with a lack of modelling and planning tools cited alongside funding and political barriers (Aldred et al., 2019). Models and tools have been developed over decades that prioritise motorised traffic, often combining walking and cycling within a residual category (Boyce and Williams, 2015). While this is starting to change (Singleton et al., 2018), ongoing barriers include a lack of data on active modes.

While all non-car modes (walking, cycling, public transport) have suffered from this traditional lack of planning tools (Curtis 2011), in England, the impact is most severe for cycling. Cycling accounts for 1% of trips, while walking is 26% (Department for Transport, 2018). Hence, existing pedestrian activity gives planners a relatively diverse and widespread starting point. Meanwhile for cycling, a ‘vehicular’ approach to provision has helped keep usage both low and dominated by the ‘strong and fearless’ (Dill and McNeil 2013), predominantly younger male adults. While cycling poses a relatively low risk to other road users (Aldred et al., 2020), cyclists themselves often experience high rates of near misses (Aldred and Goodman, 2018) and relatively high rates of injury. Women, children, and older people tend to avoid environments that require mixing with large motor vehicles on busy roads (e.g. Appleyard 2017).

In such contexts, looking at where, how, and why people *currently* cycle may provide limited insight into the characteristics of cycling if it were to become a system of mass transit. For example, if cycling is overwhelmingly male, then an approach to planning that is based on existing cycling may perpetuate that bias, given documented gender differences in trip patterns (e.g. Susilo et al., 2019), with for instance women making more ‘school-run’ trips than men. It might also be the case that those few routes that are more widely used by cyclists are popular not because of greater ‘inherent demand’ there, but rather because those are the only routes with cycling infrastructure. For these and other reasons, low-cycling contexts cannot simply extrapolate from current cycling patterns when planning for cycling growth.

Where cycling levels are low, active transport planners need a version of the ‘predict and provide’ paradigm that was so effective at enabling mode shift to driving during the second half of the 20th Century in high income countries (Naess et al., 2014). Given that transport authorities struggle to count and model current cyclists, modelling potential cyclists is a substantial challenge (Lindsey et al., 2013). However, visualising the suppressed demand represented by potential cyclists and the benefits of achieving ‘cycling potential’ might help overcome the political challenges that often prevent the building of new infrastructure in low-cycling contexts (Aldred et al., 2019).

The Propensity to Cycle Tool (PCT) was developed to make this suppressed ‘cycling potential’ visible in planning. Through this, it aims to help planners think beyond the very small and skewed amount of cycling currently existing in England and Wales. We see this skewing as an equity issue, given that (i) many inequalities (e.g. gender, age) are absent or attenuated in high-cycling settings and (ii) groups under-represented in cycling often have reduced access to other modes, such as driving. Our recently developed methodological innovations allow us to examine the distributional impacts of the PCT scenarios, in relation to carbon and health impacts of commuter cycling uptake, as well as how the distribution of cycling participation itself changes. This paper critically discusses those methods and examines what the results can tell us about possible tensions between equity, health, and environmental goals in cycling policy-making.

## 2 About the Propensity to Cycle Tool

### 2.1 Approach

The PCT approach involves calculating and visualising cycling propensity and potential^2^ at the origin-destination (OD) level (which can scale up to the national and down to the street segment level) across a range of scenarios. The underlying code is open source, and results for England and Wales are available via a freely available interactive website and data downloads (see www.pct.bike). The first national version was launched for England in 2016, providing estimates of commuter cycling potential at area, route, and network levels (Lovelace et al., 2017).

We subsequently added commuting data in Wales, and travel to school data in England (Goodman et al., 2019). More recently, the underlying model was substantially upgraded. The new approach builds on the aspatial microsimulation approach used in the Impacts of Cycling Tool www.pct.bike/ict (Woodcock et al., 2019), joining it to the PCT's spatial approach. Whereas we previously modelled at the level of origin-destination pairs (each containing aggregated data about multiple individuals), we now also model the characteristics of each individual commuter. Shifting to this individual-level approach allowed the creation of new scenarios and improved estimates of benefits, including the ability to model the distribution of these benefits across different socio-demographic groups.

### 2.2 Scenarios

The original PCT scenarios used a distance and hilliness-based function to model observed cycling, then using this model to create scenarios for new uptake.^3^ Our initial four commuting scenarios were called Government Target, Gender Equality, Go Dutch and E-bikes (Lovelace et al., 2017).

In brief:

1. **‘Government Target (Equality)’** assumes the aim of doubling cycling overall nationally is achieved. In each origin-destination (OD) pair, the number of new cyclists depends on trip distance and hilliness, with cycling increasing more in flat areas with relatively short trips. Cycling potential in this scenario assumes that within an OD pair, the likelihood of becoming a cyclist does not depend on individual characteristics, hence we have added in the ‘equality’ descriptor. Note that in this as in other scenarios, all existing cyclists are assumed to keep cycling, with the demographic biases that entails. Hence while new uptake may be more equitable, this will not be the case among those who currently cycle.
2. **‘Gender Equality’** assumes women become as likely as men are currently to cycle to work within each OD pair. Where more women than men currently cycle between a given OD pair, there is no change. Therefore, this scenario results in women having a higher cycling mode share than men do.
3. **‘Go Dutch’** assumes that commuters in England and Wales become as likely to cycle to work (allowing for trip distance and hilliness) as commuters in the Netherlands.
4. **‘E-bikes’** builds on Go Dutch but assumes people may also use electric assist bikes (pedelecs), making hilliness and distance less of a barrier. This scenario uses data from Dutch and Swiss travel surveys on electric bicycle (e-bike) usage for such trips.

We have now added the new ‘Government Target (Near Market)’ scenario. As with the Government Target (Equality) this models a doubling in cycling, but also includes new individual-level demographic predictors of cycling potential, skewing new uptake to mirror current biases. This scenario therefore differs from Government Target (Equality), Go Dutch and E-bikes scenarios, all of which use the same cycling propensity equations (based only on distance and hilliness) across all demographic groups to calculate and assign new uptake.

### 2.3 Benefits

Below and in Appendix 1 we discuss how we calculate health and carbon benefits of cycling uptake. In brief, our approach is guided by UK government appraisal methods, to which some of us have contributed. The Department for Transport has developed an Active Mode Appraisal Toolkit which seeks to calculate, monetise, and value a range of benefits from walking and cycling uptake that have previously been ignored in transport appraisal, which has tended to focus narrowly on travel time (dis)benefits to motorists.

### 2.4 Usage

As of July 2020, the PCT was used by over eighty transport authorities in England in cycle planning.^4^ The PCT underpins many Local Cycling and Walking Infrastructure Plans (LCWIPs), in which local transport authorities in England develop plans for future active travel routes (Wales has a related process under the Active Travel Act 2013). Government Target (Equality scenario; the Near Market scenario not being available until more recently) and Go Dutch are the most used scenarios.

## 3 Methods

### 3.1 Using Census 2011 data to build an individual-level synthetic population

To estimate commuter cycling potential, the PCT uses 2011 Census data on main mode of travel to work. These are the best national and geographically disaggregated data on travel patterns for England and Wales that we could access.^5^ They provide OD data on nearly 24 million commuters living in England and Wales in 2011. The OD dataset reports the number of commuters travelling between usual places of residence and main workplace locations at the level of administrative zones. We used data for Lower-layer Super Output Area (LSOA) administrative regions designed to contain a population of around 1560 individuals (average 690 commuters). The R package stplanr, developed for the PCT project (Lovelace and Ellison, 2018), was used to process the OD data.

We sought to build a synthetic population covering key demographic, social-economic and area characteristics, selecting these characteristics based on a combination of data availability, policy interest, and documented associations with travel behaviour. Our starting point of the synthetic population was a safeguarded Census dataset containing information on the age, sex and commute mode of each individual in each OD pair. In previous versions of the PCT we did our modelling at the level of the OD pair and largely only used information on commute mode share, e.g. one OD pair “A to B” might contain 3 commuters of whom 1 walked and 2 got the bus to work. In the 2019 version, we moved to the individual level, assigning to each individual their age, sex and commute mode. For example, a separate row would be created for each individual in the “A to B” OD pair (i.e. 3 rows in total), with their individual characteristics recorded as: commuter 1 is female, age 16–24, and walks; commuter 2 is female, age 16–24, and uses a bus; commuter 3 is male, age 50–64, and uses a bus.

We then enhanced these by imputing individual-level data on car ownership (binary: no car/any household car) and ethnicity (binary: white/non-white). These two demographic characteristics are both highly associated with travel behaviour and of considerable policy interest. This imputation was done probabilistically by drawing on two other safeguarded Census 2011 datasets that we commissioned specially. Because these characteristics were imputed the resulting dataset is referred to in the microsimulation literature as a ‘synthetic population’ (e.g. Lovelace and Dumont 2016). These steps are described below and summarised in a diagram in Appendix 1.

For car ownership, safeguarded data were provided indicating the number of commuters who did/did not own a car in each OD pair, stratified by mode. This was merged so that the correct total number of commuters owned a car in each OD pair, for each mode. Where there was ambiguity, the assignment was done probabilistically based on region of England/Wales, age and sex, using probabilities observed in the 5% individual-level sample released for Census 2011. For example, if in OD pair “A to B” it was known that one of the two bus commuters owned a car, car ownership would be more likely to be assigned to the older male commuter than the younger female commuter, as male gender and older age are associated with higher car ownership. For ethnicity, an identical process was used, except that ethnicity was assigned second and so could be assigned based on region, age, sex and car ownership.

Finally, based on home and work location, we merged in the following area-level variables:

• Income Deprivation of the home LSOA, using the Index of Multiple Deprivation data for England and Wales. We ranked LSOAs into quintiles for income deprivation for England and Wales separately.
• Urban-rural status and sparsity of the home LSOA.
• Estimated distance and gradient of the ‘fastest’ routes between the home LSOA and work LSOA, using CycleStreets.net (see Lovelace et al., 2017).

Income deprivation was selected as a measure of socio-economic position (income information is not available at individual level in the Census) and urban/rural status and sparsity as being standard geographic measures of land use available at the LSOA level.

### 3.2 Creating scenarios of cycling potential: summary of existing four scenarios

The original scenarios estimated cycling potential as a function of two variables: route distance and route hilliness (Lovelace et al., 2017). We used logistic regression applied at the individual level, modelling the relationship between the proportion of commuters cycling (the dependent variable) and the fastest-route distance and route gradient (the two explanatory variables). Our equations included terms to capture the non-linear impact of distance on the likelihood of cycling, and ‘interaction’ terms, as the impact of trip distance varies according by hilliness. We also developed equations to estimate commuting mode share among groups with no fixed workplace. This model of baseline propensity to cycle formed the basis of the scenarios Government Target (Equality), Go Dutch and E-bikes.

We focused on distance and hilliness as strong predictors of cycling, and as expressing relationships less amenable to change than, for example, gender and cycling, which has very different relationships in high- and low-cycling contexts (Aldred et al., 2016a). We focused on the more direct ‘fastest’ routes as these are where the cycling potential is likely to be greatest if appropriate infrastructure is constructed.

### 3.3 Creating scenarios of cycling potential: Near Market scenario

Using the new synthetic population, with additional individual characteristics, we created a new ‘Government Target (Near Market)’ scenario. Both Government Target (Equality) and Government Target (Near Market) scenarios model doubling of cycling trips nationally, corresponding to the national target for 2025^6^ (Department for Transport, 2017).

Specifically, as in the earlier scenarios, we estimated propensity to cycle among 19 million English and Welsh commuters with a CycleStreets fast route distance of <30 km between their home and workplace. We did this by fitting logit regression models using predictor variables to capture the effect of distance and gradient. The difference was that in the Near Market scenario we took account of a wider range of variables:

1. Region (11 regions: the 10 standard regions of England and Wales, subdividing London into Inner and Outer London),
2. Sex (binary),
3. Age category (16–24; 25 to 34; 35 to 49; 50 to 64; 65 to 74; 75+),
4. Ethnicity (white, non-white),
5. Having a household car (binary),
6. Fifths of income deprivation,
7. Urban-rural status (Urban major conurbation; Urban minor conurbation; Urban city and town; Rural town and fringe; Rural village and dispersed),
8. A sparsity index, identifying the sparsest 5% of areas in terms of population (binary).

We did this by (i) stratifying by region, sex, and broad age band (16–49, and 50+) and then (ii) entering the other variables into the model as predictors. In total we modelled baseline propensity to cycle through 44 regression models (11 regions * male/female * 2 age categories). This process of stratification allowed the predictor variables’ importance to vary according to age, sex, or region. For example, the deterrent effect of longer distance is greater in women and in older people than in young men; and car ownership is less strongly (negatively) associated with cycling in London than in other regions of England and Wales. Further details can be found in PCT User Manual C1,^7^ which also contains further details and equations related to the previous four scenarios.

Besides these 19 million commuters, a further 2.1 million had no fixed workplace. Their propensity to cycle was modelled as a function of the weighted average for individuals in their OD pair, the same method also used for the other scenarios. A further 2.9 million individuals lived further than 30 km from their workplace or worked overseas and were assumed to have no increase in cycling.

Finally, to enhance comparability between the two Government Target scenarios, the overall increase in any region was adjusted for Government Target (Near Market) to be equal to the already existing Government Target (Equality) scenario. In other words, the regional increase is determined in both versions only by distance and hilliness, while for Government Target (Near Market) the distribution of cycle commuting uptake within regions is also affected by the demographic variables listed above.

### 3.4 Modelling mode shift

To estimate health and carbon benefits, we needed to estimate which modes switched to cycling. For instance, if trips shift from walking to cycling, there is a reduction in physical activity to be included when calculating health impacts of scenarios; while carbon reduction benefits stem only from reductions in driving (assuming public transport continues to run).

For our original scenarios, new cyclists were initially generated for each OD pair, based on distance and hilliness (or for Gender Equality, based on the proportion of men already cycling to work). For each OD pair, we then allocated those new cyclists to have come from other modes based on the current mode split. So, for instance, if in the 2011 Census 1/3 of all non-cyclist commuters travelling between A and B currently walked and 2/3 currently used the bus, then likewise 1/3 of the new cyclists would be ex-walkers and 2/3 ex-bus users. Where much existing commuting is by walking or public transport (as for commutes within dense city centres), new cyclists will often come from those modes; while in OD pairs where car is dominant, many new cyclists will come from the car.

By contrast, the Near Market scenario estimates how cycling uptake varies between commuters within an OD pair as a function of their individual characteristics (sex, age, ethnicity, car ownership, income and other distributional factors). Current commuting mode is not directly used to predict cycling uptake. However, to the extent that current commuting mode is associated with these other individual characteristics, there are different relative mode shifts for different modes within the same OD pair. For example, people without a household car are less likely to commute by car and in the Near Market scenario have a higher cycling potential.

### 3.5 Calculating physical activity

Additional physical activity was calculated for new cyclists based on the average number of cycle commute trips per week * trip duration * physical activity energy intensity. In cases where a trip was previously walked, the displaced physical activity was calculated using a similar approach. Our units for calculating physical activity were marginal Metabolically Equivalent Tasks (mMETs) hours per week. An mMET is a measure of the intensity on activity above resting (Ainsworth et al., 2011; Woodcock et al., 2019).

Because most cycle commuters do not cycle commute twice a day for every working day of the year, average number of cycle commute trips per cyclist per week were estimated, stratified by age and sex, from the English National Travel Survey (Cornick et al. 2020).

Going beyond work presented in Lovelace et al. (2017), we modelled speed and intensity (mMET rate per hour) as a function of gradient. Below we summarise the method, with further details given in Appendix 1.

We used the equation from di Prampero et al. (1979) that calculates the power required by a cyclist to move based on road resistance, wind resistance, and gravity. We developed a decay function for uphill moving speed with incline, based on published studies (Costa et al., 2015; Sperlich et al., 2012). We fitted this decay function using linear regression, with speed as the outcome and the square root of incline as the predictor.

The PCT is currently based on average gradients, i.e. a gradient of 1.5% means an average uphill gradient of 1.5% in one direction, and downhill the other. We assumed that energy expenditure and speed when going downhill was equal to energy expenditure and speed when travelling on the flat.

As our primary focus was on cycling, for walking we made the simplifying assumption that the relative effort and speed penalty of walking uphill were directly proportional to the relative effort and speed penalty of cycling uphill. We adjusted values to maintain a mean walking value of 3.6 mMET.

We assumed that an e-bike halved the additional effort required uphill, in line with our previous observation that the deterrent effect of hills for e-bike-owners was around half that of non-e-bike owners (Lovelace et al., 2017). We further assumed that cycling on the flat was 1.8 mMET lower intensity on an e-bike than on a bicycle. Together this approximately generated the average e-biking mMET of 3.5 that has been reported in the literature. We assumed the relative speed penalty of travelling on a hill is smaller than for a traditional bike.

Based on the above we calculated average mMET rates and speeds for cycling, walking, and e-biking for routes of different gradient. For the average commuter gradient in 2011 of 1.5% this gives a speed of 13.9 km/h and mMET value of 5.39. This is close to the WHO Health Economic Assessment Tool (HEAT) average estimates.

### 3.6 Modelling health impacts

Health benefits were calculated as change in premature deaths and Years of Life Lost (YLLs), building on an approach we and others had developed for the UK Department for Transport (Department for Transport, 2020). Cycling trip duration was estimated as a function of the ‘fastest’ route distance and average cycling speed.

A relative risk of 0.9 was used for an increase in cycling or walking 8.75 mMETh per week (roughly equivalent to 150 min of moderate intensity exercise). The impact fraction was calculated by scaling these relative risks to the power of the modelled weekly mMEThs vs 8.75. For example, if an individual is modelled to have an increase of 1.2 mMETh, the relative risk applied would be (0.9^(1.2/8.75)) = 0.986, or a 1.4% decrease. The total possible relative risk was capped at 0.55 (45% decrease) for cycling and 0.70 (30% decrease) for walking.

We assigned a mortality rate to each individual based on their age, sex, and local authority, using background mortality rates for each local authority in England and Wales in 2016.

The net change in the number of deaths avoided for each OD pair was estimated as the number of deaths avoided due to increased cycle commuting minus the number of additional deaths incurred due to reduced walking.

We converted our estimate of the net number of deaths avoided into an estimate of the number of YLLs avoided. We did this by using Global Burden of Disease data from 2017 in England and Wales to estimate the average YLL loss per death. This was done separately by age group, sex and region. As recommended in UK appraisal methods, future benefits were discounted by 1.5% per year. The monetary value of the mortality impact was calculated by multiplying the number of YLLs avoided by £57,965, the value of a statistical life year used by Department for Transport, in 2010 prices. Our results are presented for deaths in a single year (noting that YLLs saved from a death occur over many years in the future).

### 3.7 Sickness absence

The Department for Transport's Transport Appraisal Guidance (TAG) on calculating health impacts of transport schemes covers the economic impacts of changes in sickness absence alongside health economic benefits from physical activity. Building on this guidance, we estimated the economic value of reduced sickness absence using an approach similar to that used to estimate the reduction in mortality. We used an identical approach to calculate the change in mMEThs. Based on TAG values we used a 0.25 relative reduction in short-term sickness absence associated with an increase in cycling or walking of 8.75 mMETs/week. The relative risk was capped at 0.50 (50% decrease) for both cycling and walking.

Average hours of sickness absence are a function of sickness absence rate and total working hours. These both vary by sex, age, and region. We therefore calculated age and sex-specific average annual hours of sickness absence for regions in England and for Wales (ranging from 8.2 h/year for men aged 16–24 in the East Midlands to 69.9 h/year for men aged 50–64 in Wales).

As with premature deaths, we calculated the reduction in sickness absence due to increased cycling and then subtracted the increase in sickness absence due to decreased walking.

Finally, we multiplied the net change in annual sickness hours by mean hourly salary costs. We scaled this figure to vary by region, using 2018 median salaries (ranging from £17.16 in the North East to £24.15 in London).

### 3.8 Carbon emissions

When comparing each scenario to baseline, we estimated the reduction in transport carbon dioxide (CO2) emissions as follows:

Change in CO2-equivalent emissions (in kg) per year = Change in no. car drivers * former distance travelled by former car drivers * mean cycle commute trips per cyclist per week * 52.2 * CO2-equivalent emissions (in kg) per kilometre.

Their average former distance was assumed equal to the new ‘fastest-route’ distance travelled by the cycle commuters. The average CO2-equivalent emission per kilometre car driving was taken as 0.182 kg, the 2017 value for an ‘average’ car of ‘unknown’ size in the UK government's carbon conversion factors. Results were monetised using Department for Transport recommended values.

## 4 Results

Below we present selected results focusing on the impact of our methodological innovations used in the new model, specifically hilliness and demographic differences in uptake and subsequent benefits.

### 4.1 Characteristics affecting uptake and benefits, by demographic group

Table 1 presents the underlying trip characteristics, baseline mode shares for walking, driving, and cycling, by demographic group.^8^ The trip characteristics illustrate the extent to which some demographic groups make more or fewer ‘cyclable’ trips (in terms of distance and hilliness), and which groups are most likely to shift from the car versus other modes. For instance, rural trips are substantially longer and hillier than trips made by urban residents. In terms of mode share, the richer groups are substantially more likely to be car commuters at baseline, and less likely to be cycle commuters. Lower income groups both have shorter, more cyclable commutes and are more likely to already cycle. By contrast, while women are currently much less likely to cycle to work than are men, they tend on average to make significantly shorter (albeit slightly hillier) trips.

**Table 1:** Trip characteristics and baseline mode share by demographic group, all commuters.

		Mean commute trip length (km), among trips <30 km^a^	Mean commute hilliness gradient (%), among trips <30 m^a^	% commuters walking (baseline)	% commuters who are car driver^b^ (baseline)	% commuters who are cyclists (baseline)
Whole sample		8.8	1.9%	10.9%	60.7%	3.1%
Sex	Male	9.6	1.8%	8.4%	63.3%	4.4%
	Female	8.1	1.9%	13.7%	57.9%	1.7%
Age	16 to 24	7.8	1.9%	18.0%	41.1%	2.9%
	25 to 34	9.1	1.8%	11.0%	54.3%	3.7%
	35 to 49	9.1	1.9%	9.0%	66.6%	3.3%
	50 to 64	8.6	1.9%	9.7%	68.4%	2.5%
	65+	8.0	1.9%	11.2%	65.1%	2.1%
Ethnicity	White	8.8	1.9%	10.8%	63.1%	3.3%
	Non-white	8.6	1.6%	11.5%	42.9%	1.9%
Household car	1 or more cars	9.1	1.9%	8.7%	68.0%	2.6%
	No car	6.8	1.7%	25.1%	13.3%	6.2%
Income deprivation	Fifth 1 (poorest)	7.4	1.8%	14.1%	48.4%	3.2%
	Fifth 2	8.2	1.8%	12.7%	54.2%	3.4%
	Fifth 3	8.9	1.9%	11.0%	61.4%	3.2%
	Fifth 4	9.6	1.9%	9.0%	67.9%	2.9%
	Fifth 5 (richest)	9.9	1.9%	8.4%	69.2%	2.9%
Urban/rural	Rural	11.6	2.1%	7.6%	77.0%	1.9%
	Urban	8.3	1.8%	11.6%	57.3%	3.4%

a In our model no mode shift to cycling occurred for commutes ≥30 km.

b Drivers only, i.e. not including car passengers, for whom a switch to cycling entails health but no carbon benefits in our model.

**Table 2:** Number of new cyclists and total mode share for cycling, per scenario, by demographic group.

		No. comm-uters	Baseline		Government target: Near market		Government target: Equality		Gender Equality		Go Dutch		E-bikes	
			N cyclists	% cycling	N new cyclists	% cycling^a^	N new cyclists	% cycling^a^	N new cyclists	% cycling	N new cyclists	% cycling^a^	N new cyclists	% cycling^a^
Whole sample		23,903,549	744,459	3.1%	715,619	6.1%	711,673	6.1%	379,881	4.7%	3,774,751	18.9%	5,306,421	25.3%
Sex	Male	12,467,760	544,895	4.4%	521,358	8.6%	325,535	7.0%	–	4.4%	1,677,047	17.8%	2,418,273	23.8%
	Female	11,435,789	199,564	1.7%	194,262	3.4%	386,138	5.1%	379,881	5.1%	2,097,703	20.1%	2,888,148	27.0%
Age	16 to 24	3,237,168	94,487	2.9%	89,632	5.7%	106,856	6.2%	56,566	4.7%	577,862	20.8%	795,611	27.5%
	25 to 34	5,538,697	203,555	3.7%	185,172	7.0%	165,239	6.7%	86,120	5.2%	852,114	19.1%	1,201,813	25.4%
	35 to 49	8,650,594	286,156	3.3%	277,449	6.5%	245,259	6.1%	134,339	4.9%	1,297,370	18.3%	1,837,546	24.5%
	50 to 64	5,804,849	145,953	2.5%	148,982	5.1%	173,152	5.5%	92,688	4.1%	931,783	18.6%	1,310,908	25.1%
	65+	672,241	14,308	2.1%	14,385	4.3%	21,168	5.3%	10,168	3.6%	115,622	19.3%	160,544	26.0%
Ethnicity	White	21,050,896	689,034	3.3%	660,368	6.4%	613,538	6.2%	341,689	4.9%	3,253,236	18.7%	4,600,125	25.1%
	Non-white	2,852,653	55,425	1.9%	55,251	3.9%	98,135	5.4%	38,192	3.3%	521,514	20.2%	706,296	26.7%
Household car	1 or more cars	20,703,404	544,801	2.6%	542,414	5.3%	594,789	5.5%	318,352	4.2%	3,150,270	17.8%	4,473,058	24.2%
	No car	3,200,146	199,658	6.2%	173,206	11.7%	116,885	9.9%	61,529	8.2%	624,480	25.8%	833,362	32.3%
Income	Fifth 1 (poorest)	4,076,504	130,750	3.2%	132,009	6.4%	143,782	6.7%	64,417	4.8%	795,105	22.7%	1,074,781	29.6%
Deprivation (area level)	Fifth 2	4,872,476	163,999	3.4%	155,366	6.6%	157,254	6.6%	80,691	5.0%	843,191	20.7%	1,162,164	27.2%
	Fifth 3	5,060,155	162,602	3.2%	152,764	6.2%	149,731	6.2%	83,252	4.9%	787,422	18.8%	1,109,086	25.1%
	Fifth 4	4,996,073	143,772	2.9%	138,149	5.6%	134,491	5.6%	76,691	4.4%	698,810	16.9%	1,010,066	23.1%
	Fifth 5 (richest)	4,898,341	143,336	2.9%	137,332	5.7%	126,416	5.5%	74,830	4.5%	650,222	16.2%	950,325	22.3%
Urban/rural	Rural	4,103,067	77,649	1.9%	71,303	3.6%	79,382	3.8%	44,367	3.0%	401,028	11.7%	637,060	17.4%
	Urban	19,800,482	666,810	3.4%	644,316	6.6%	632,291	6.6%	335,515	5.1%	3,373,722	20.4%	4,669,361	26.9%

a In all cases, mode share for cycling comes from adding the N new cyclists to the N cyclists at baseline, i.e. it is total mode share.

Alongside baseline health data and hourly salary (see Table 3), the trip characteristics and mode share will also determine the benefits per group of cycling uptake. For instance, those aged 16–24 are substantially more likely to walk to work than any other group. Thus, any switched trip among this group is more likely to come from walking, a switch that results in a net health loss. To give another example, white people are relatively likely to drive to work, compared to non-white people. This means that any trips switched among the former group are more likely to result in a carbon benefit.

**Table 3:** Baseline disease burden measures by demographic group, all commuters.

		Mean annual mortality rate, per 1000 commuters	Mean YLL per death	Mean annual YLLs, per 1000 commuters (i.e. mortality rate * YLLS)	Mean annual hours of sickness per commuter	% commuters over age 50 years
Whole sample		2.28	33.6	76.6	31.3	27%
Sex	Male	2.82	33.6	94.8	29.6	27%
	Female	1.70	33.7	57.3	33.2	27%
Age	16 to 24	0.24	43.1	10.3	18.0	0%
	25 to 34	0.51	39.5	20.1	25.2	0%
	35 to 49	1.44	33.2	47.8	30.1	0%
	50 to 64	4.68	25.4	118.9	46.5	100%
	65+	16.92	16.8	284.3	30.2	100%
Ethnicity	White	2.37	33.4	79.2	32.0	29%
	Non-white	1.64	35.3	57.9	26.6	16%
Household car	1 or more cars	2.35	33.3	78.3	31.9	28%
	No car	1.83	35.5	65.0	27.5	18%
Income deprivation	Fifth 1 (poorest)	1.98	34.5	68.3	31.1	22%
	Fifth 2	2.10	34.2	71.8	30.3	24%
	Fifth 3	2.28	33.7	76.8	31.0	27%
	Fifth 4	2.46	33.1	81.4	32.1	30%
	Fifth 5 (richest)	2.53	32.8	83.0	32.2	31%
Urban/rural	Rural	2.71	32.4	87.8	33.8	34%
	Urban	2.19	33.9	74.2	30.8	26%

Note that background mortality rate, YLL per death and annual hours of sickness are varied as a function of region, age and gender, but not according to the other demographic characteristics shown here. Our model therefore does not capture additional differences by other characteristics such as income. The sickness absence calculation requires mean hourly salary, and we only used a regional figure for this; hence it does not capture additional demographic variation; for instance, the large gap between male and female or white and non-white earnings.

### 4.2 Mode share in different scenarios

The discussion above highlights differences in trip characteristics by demographic group. Building on this, Table 2 shows the number of new cyclists and subsequent mode share by demographic group for each scenario. In all scenarios, the number of new cyclists is affected by the types of trip made by that type of individual, specifically trip distance and trip hilliness. Hence, under Government Target (Equality) and Go Dutch, there are more new female cyclists than new male cyclists because women tend to make shorter trips.

In the case of Go Dutch, this is enough to yield an overall higher mode share for women cycling. By contrast, in Government Target (Equality) scenario men's mode share remains higher because, given our simplifying assumption that all existing cyclists keep cycling, new uptake makes up a smaller percentage of total cyclists, and existing cycling (see baseline) is highly gender-skewed. For the Government Target (Near Market) scenario, the number of new cyclists is based on cycling propensities related to individual characteristics as well as trip characteristics. As these individual cycling propensities mirror baseline cycling propensities, the gender skew remains even among the new cyclists.

### 4.3 Health, health economics, and carbon impacts

Table 3 presents the data on mortality rates, YLLs, and annual hours of sickness absence used in the model. These alongside the trip characteristics discussed above feed through into the benefits gained per group. Uptake and benefits may pull in different directions. For instance, rural trips are longer and hence less likely to shift to cycling; however, they are also currently relatively car-dependent, so per trip switched, carbon benefits are on average higher in rural than in urban areas. Table 3 illustrates how age is the major driver of health benefits, given the sharp increase in mortality rate in the older age groups, only partly counteracted by the lower YLLs per death. Differences in average ages mainly explain other differences such as the urban/rural comparison (rural populations being older on average) or the differences by income deprivation (people in affluent areas being older on average). To illustrate this, Table 3 also shows the proportion of commuters in each demographic group aged over 50.

Table 4 below presents total health, health economic and carbon impacts across all commuters, for the five scenarios compared with baseline, and for baseline (compared with a hypothetical no cycling). In broad terms, the scenario benefits largely track the change in uptake. The E-bikes scenario produced the largest gains in health, with notably larger reduction in CO_2_ emissions than the other scenarios, due to the longer trips cycled. The Gender Equality scenario produces relatively low health economic benefits, due (i) to the relatively low increase in cycling, with just under 400,000 compared to just over 700,000 in the two Government Target scenarios, and (ii) all the new cyclists being women. While more women cyclists is good for equity, it leads to comparatively lower health and carbon benefits, based on (i) women's lower baseline mortality risk than men, (ii) women's lower commute travel distances than men, and (iii) women's lower propensity to commute by car.

**Table 4:** Total health, health economic and carbon impacts, across all commuters (N = 23,903,549).

	Baseline (relative to no cycling)	Scenarios (changes relative to baseline)				
		Government Target: Near Market	Government Target: Equality	Gender Equality	Go Dutch	E-bikes
Deaths averted per year	198	211	217	74	939	1062
YLLs averted per year	5,454	5,830	5,624	1,922	24,273	27,520
Reduction in person-years of sickness absenteeism per year	1,878	1,981	2,107	1,068	9,910	11,869
Millions of pounds of health economic benefit (YLL + sickness absence) per year	416	442	436	167	1,923	2,211
Reduction in thousands of tonnes of transport CO2 equivalent per year	104	115	112	43	496	859

Appendix 2 shows how the results in Table 4 are distributed across demographic groups for all scenarios; with the size of the benefit in each group being a function in part of the number of people in that group. To facilitate a comparison of e relative benefits across group, Table 5 illustrates the benefits obtained in the Go Dutch scenario per new cyclist (per commuter is provided in Appendix 2). The per-commuter benefit in other scenarios was similar.

**Table 5:** Health, health economic and carbon impacts in the Go Dutch scenario, =per million new cyclists.

		Deaths averted per year	YLLs averted per year	Reduction in person-years of sickness absenteeism per year	Millions of pounds of health economic benefit (YLL + sickness absence) per year	Reduction in thousands of tonnes of transport CO_2_ equivalent per year
Whole sample		249	6430	2625	509	131
Sex	Male	348	9031	2736	665	162
	Female	169	4351	2537	385	107
Age	16 to 24	21	919	1246	118	81
	25 to 34	52	2058	2087	231	119
	35 to 49	153	5083	2566	428	146
	50 to 64	516	13137	4076	972	153
	65+	1756	27270	2465	1704	138
Ethnicity	White	259	6640	2684	523	137
	Non-white	184	5127	2261	426	96
Household	1 or more cars	272	7014	2839	553	153
car	No car	133	3489	1548	290	24
Income	Fifth 1 (poorest)	194	5170	2396	425	96
deprivation	Fifth 2	213	5616	2375	452	111
	Fifth 3	248	6423	2572	507	133
	Fifth 4	293	7454	2898	581	159
	Fifth 5 (richest)	315	7937	3004	615	169
Urban/rural	Rural	379	9471	3404	718	214
	Urban	233	6069	2533	485	122

Values, and their distribution across characteristics, are similar in other scenarios.

In Table 5 we see under the Go Dutch scenario substantial variation based on the factors previously discussed. Although richer areas have fewer cyclable trips (average commute distance of 9.9 km for the top income fifth compared to 7.4 km for the poorest), those who do switch to cycling on average will each generate more health and carbon benefits, because (i) each trip cycled is longer on average, so generates more physical activity energy expenditure, (ii) this group is older on average, so obtains more health benefits from physical activity, and (iii) trips are relatively likely to come from the car, hence more likely to generate carbon benefits.

In poorer areas, although trips are more likely to switch to cycling, these (shorter) trips are less likely to come from the car, reducing carbon benefits; and are more likely to come from walking (the baseline mode share of walking is 14.1% for the poorest fifth compared to 8.4% for the richest fifth), which generates a net health loss. The result is that the economic benefit *per new cyclist* is much greater in richer areas e.g. 315 deaths and 169 thousand tonnes of CO_2_ averted per million cyclists for the richest fifth, versus 194 deaths and 96 tonnes CO2 for the poorest fifth. However, because fewer people switch to cycling in richer areas, the impact *per commuter* is more similar, e.g. 42 deaths and 22 thousand tonnes of CO_2_ averted per million commuters for the richest fifth, versus 38 deaths and 19 tonnes CO_2_ for the poorest fifth. In summary: greater uptake is likely in poorer areas, but each switching cyclist in poorer areas generates lower health and carbon benefits, so overall the benefits are similar.

Comparing by gender and age group, differences in YLLs and deaths averted stem from major differences in background mortality risk, which will feed through into other group differences (e.g. rural residents may have older age profiles than urban residents). As older people and, to a lesser extent, men have higher background mortality risk than younger people and women, getting the former groups cycling generates more health benefits per individual taking up cycling. For men, this is further increased by their higher propensity to travel longer distances (9.6 km average commute distance versus 8.1 km for women).

The difference by age is particularly large for deaths, and somewhat smaller for YLLs because each death corresponds to a larger number of YLLs for a younger person. For sickness absence the effect is less marked and peaks in age 50–64, reflecting the fact that this group works more hours per year than commuters age 65+.

### 4.4 Hilliness

Hilliness, as well as distance, affects the likelihood and health benefits of cycling. As described in the Methods section, our previous model included the impact of hilliness on uptake, and our new model additionally incorporates the impact of hilliness on the health benefits. In England and Wales’ eight hilliest areas (average slope 4% or more), modelled cycling uptake is relatively low, with a 7.6% mode share under the Go Dutch scenario. By contrast, this figure is three times higher in the 43 flattest authorities (average slope less than 1%). Once hilliness is additionally included in the health impact model, however, the difference between the hillier and the flatter areas narrows somewhat in terms of health impact. Specifically, when only cycling uptake is considered (as in the original version of the PCT), the overall population health benefits in hilly areas are around a third of that of flat areas. Once the additional health benefits of hilliness are factored in, this rises to half as much (Table 6).

**Table 6:** Effect of calculating impacts of hilliness on health benefits, for the Go Dutch scenario^a^.

Average Local Authority slope %	Number of local authorities	Commuting population	Percentage cycling	YLLs gained per 1000 population of commuters, not factoring in hilliness	YLLs gained per 1000 population of commuters, factoring in hilliness
0 to 0.99	43	2,854,898	21%	1.41	1.25
1 to 1.99	162	11,724,801	17.3%	1.12	1.10
2 to 2.99	100	6,393,307	13.3%	0.82	0.89
3 to 3.99	35	2,454,319	10.5%	0.65	0.77
4 to 4.84 (max)	8	476,224	7.6%	0.45	0.57

a Average calculated for trips <10 km.

## 5 Discussion

### 5.1 Summary of findings

Recent updates to the PCT have incorporated an individual-level model of cycling uptake. This enabled calculation of more refined outputs for five different scenarios, including the new Near Market scenario. The results estimate cycling uptake, mode share, health and carbon impacts for each scenario, at area, route, route network and individual levels. Using this model, we have shown both overall differences in impacts between the scenarios, distributional differences by a range of demographic variables, and the impact of including hilliness within the calculation of health benefits of cycling. A key finding is that while cycle commuting in England and Wales varies substantially by gender and ethnicity, women and non-white people tend to make more ‘cyclable’ commute trips (in terms of distance and hilliness). A key finding of this paper is that established appraisal methods (including our own in PCT) assign less value to these shorter trips.

### 5.2 Meaning of our findings

The Near Market scenario can be understood as an indication of how commuter cycling in England and Wales might look, demographically, if the Government Target of doubling cycling were achieved without significant broadening of cycling's demographic appeal. Under this scenario, despite the shorter and hence more cyclable trips made by women and non-white people, it remains dominated by white men. The Government Target (Equality) scenario is more diverse but still demographically skewed, due to the inequalities among existing cyclists, who comprise 50% of all commuter cyclists in both Near Market and Equality Government Target scenarios.

We have assumed that current cyclists (based on 2011 data) continue to cycle and that any increase in cycling is in addition to these cyclists. This assumption means that small increases in cycling lead to only a small change in the demographic composition of the cycling population. In reality, there is considerable ‘churn’ in cycling. However, it is likely that policies to increase cycling will require reducing churn (catering better for existing cyclists) as well as increasing the number of new cyclists. From 2001 to 2011 we did not see improvements in the gender and age mix of commuter cycling in those places where cycling levels increased (Aldred et al., 2016). Thus, we consider our simplifying assumption is reasonable, and that demographic skew amongst cyclists is unlikely to decrease markedly while cycling levels remain relatively low.

The more ambitious scenarios mean, however, that most cyclists are ‘new’. This allows the ‘naturally’ more cyclable trips to dominate, with women and non-white adults having higher mode share, despite starting from a lower baseline. Indeed, it is difficult to imagine a high-cycling scenario in which very large demographic inequalities remain, especially among numerically large groups such as women and older people (Aldred et al., 2016). Thus, cycle planning should have a strong equity focus, in addition to supporting existing cyclists. This would include going beyond focusing on the simple commute, given the dominance of women in the school run (often as part of a trip chain), and the different distribution of schools compared to workplaces (Goodman et al., 2019). As women show particularly strong preferences for cycling environments separated from motor traffic (Aldred et al., 2016b) routes to such destinations need to involve high quality cycleways.

By contrast, the under-representation of cyclists among commuters living in the wealthier areas and (more so) car owners persists in all scenarios. These groups are likely to make longer and less cyclable commutes. Yet although longer trips are less likely to be switched to cycling, they do generate greater health benefits and (if switched from driving) higher carbon benefits. Hence groups such as men, who make longer trips and are more likely than women to drive, generate relatively high carbon benefits per commuter switched. Because of their higher baseline mortality risk and those longer trip distances, alongside their low walking at baseline compared to women, a man switching to cycling experiences greater health benefits on average than would a woman. At population level, this implies greater societal health economic benefit from getting 1,000 men cycling, compared to 1,000 women.

Returning to hilliness, this too has interesting implications (including for hillier countries). Where national allocation of resources to cycling is considered, it would be easy to ignore or marginalise hilly areas that have lower cycling potential. However, while hilliness suppresses uptake, it also increases health benefits from uptake. Finally, the urban/rural divide narrows in the more ambitious scenarios, highlighting the greater suppression of cycling demand in rural areas.

### 5.3 Strengths and limitations

Strengths of this work include the individual-level modelling that enables analysis of demographic variation in uptake and benefits obtained. The analysis of hilliness allows quantification of the extra health benefits obtained by getting more people cycling in hillier areas, supplementing our existing work showing the possible impact of e-bikes on uptake in such areas. The sophisticated propensity modelling developed here at a national scale for areas, routes, and networks goes beyond most work in the area. The most comparable work we know of has been done by Transport for London (TfL 2016), who have created a similar active travel potential model for all trip purposes using the more advanced data available to planners in that city-region (but not more widely or nationally available).

A major strength of the PCT approach is that the tool, the data, and the code are freely available to the public, consultancies, and local authority transport planners, reducing the information gap between diverse stakeholders in the decision-making process. This ‘open access’ approach transport modelling goes beyond the highly technical approach of transport modelling software products such as SUMO and MATSim that are open but only accessible to experts working in the field (Lovelace et al. 2020). The open source nature of the tool means that the model outlined in this paper could be extended, e.g. by alternative ‘impedance functions’ (Martínez and Viegas 2013; Levinson and King 2020) and inclusion of additional explanatory variables in the scenarios of change (e.g. Larsen et al., 2013).

The outputs of the PCT are provided as open access datasets, and can be combined at the local level with other data such as infrastructure, obesity, public transport, new developments. One national example of this combination is the Department for Transport and Sustrans funded Rapid Cycleway Prioritisation Tool (freely available, at https://www.cyipt.bike/rapid/). This has been used to prioritise the location of ‘pop-up’ cycleways where there is both sufficient road width and high cycling potential in the context of COVID-19 (Lovelace et al., 2020). Furthermore, users can use the pct R package to download data and reproduce the findings (see https://cran.r-project.org/package=pct).

As with all models ours has many limitations and we can only highlight some of them. We only use a subset of demographic factors to calculate health and absenteeism benefits. This means that absenteeism benefits cannot be meaningfully disaggregated according to other demographic characteristics (e.g. ethnicity), and that we are for instance underestimating the physical activity benefits from take-up of cycling among lower income groups within a given local authority.

Health benefits covered here are those modelled in the PCT; physical activity and sickness absence. Other health impacts might relate for instance to air pollution, noise pollution, and injury risk. Health impacts of mode shifts to cycling are in countries such as the UK dominated by change in physical activity (Woodcock et al., 2013; Muller et al., 2015). Changes in injury risk depend not just on mode shift but local factors, notably on the kind of infrastructure is built, and this is not specified in the PCT. Over the longer term and a bigger geography, reductions in carbon emissions will also benefit health, although the reductions here are small.

Our health model (following UK appraisal methods) is relatively naïve, e.g. not accounting for longer term health benefits and only including mortality (Mytton et al., 2017). However, we have included several aspects not typically captured in UK appraisal notably, hilliness, local authority specific mortality, and detailed age and sex information.

We have assumed that travel distances are fixed. While reducing travel distances can play a major role in reducing transport related emissions, our results show that substantial mode shift can be achieved with current travel distances. The infrastructure and supporting interventions required to achieve this are likely quicker than changes in land use (Ahmad et al. 2019).

We are limited by our main dataset, from 2011 and only including commuter trips, which excludes many older adults, those out of work, and biases towards men.

### 5.4 Research and policy recommendations

Our study highlights the extent of England and Wales’ cycling potential and the large corresponding health and carbon benefits from achieving some or all of this potential uptake.

This paper highlights that many of the groups currently under-represented in cycling in England and Wales make commute trips that are relatively conducive to cycling. All else being equal, one might expect women to cycle more than do men. In environments where cycling is culturally normalised and experienced as safe, such as the Netherlands, women currently cycle more than men; and within England there is a strong correlation between levels of cycling and gender equity in cycling (Aldred et al., 2016). The analysis here suggests that in England and Wales too, women may be more ‘natural’ cyclists than men based on trip distances (McQuaid 2009). Our analysis provides further evidence on how cycling can help realise multiple co-benefits, but goes beyond previous work by showing that different policy goals (carbon, health, equity, efficiency, etc.) may imply differing priorities in terms of where (and who) to target resources. Finally, hilly areas should not automatically be overlooked for investment based on their lower uptake, particularly where health benefits of cycling are prioritised, and/or e-bike usage is growing.

Our findings have implications for ‘active mode appraisal’. Traditionally, transport appraisal relied on time savings for motorists to drive decision-making, and inclusion of health and carbon impacts are seen as redressing the balance and creating fairer appraisal methods. However, especially with more sophisticated demographic representation, this has the potential to act as a barrier to equity goals, and, more sophisticated demographic representation could make this worse. Sickness absence calculations rely on earnings, and carbon reduction benefits stem from trip switching from driving: both will tend to place more value on cycling uptake that perpetuates existing demographic skews in England and Wales.^9^

Perhaps more surprisingly, calculations of health benefits from physical activity (accounting here for most health economic benefits) can also be anti-equity, assigning here more value to trip switching by men, white people, and people in more affluent areas (the latter two effects largely reflecting the fact that these groups are older).

While the inequitable metrics reflect real differences in life expectancy and carbon emissions, they are also a result of which trips and which outcomes are considered by the PCT and English appraisal methods. For example, someone without a car will not generate carbon savings but might get a transformed ability to access a wide range of locations, while a young woman might not get as many YLL savings but still might experience other benefits of exercise e.g. mental health.

Cycling may also be seen as a good in itself that should be accessible to all and this is likely to be increasingly true as cycling rates increase.

For both these reasons, there is an argument to include equity in commuter cycling levels as an independent criterion, to ensure that the inclusion of health and carbon benefits does not encourage inequitable policy-making. Practical steps to increase equity might include ensuring that cycling network plans cover school as well as commute trips, better meeting women's and children's journey needs, and conducting spatial equity assessments of planned bike networks to ensure that different community needs are served. We have not included the potential for cycling to open up new commuting opportunities (most notably for those without a car), which should be accounted for in specific appraisal of schemes, and which could increase equity.

## 6 Conclusion

We have shown the potential of new methods to improve tools for modelling cycling potential and subsequent impacts at multiple scales. Presented in a publicly available web application and supported by the UK's Department for Transport, we have also demonstrated how cycling uptake models can inform policies and prioritise interventions on local, regional and national scales. Using individual-level modelling allows the identification of differential impacts, as well as changes to mode split for different demographic groups under different scenarios. We found that many people in under-represented groups have high cycling potential based on trip characteristics but, due to the focus of appraisal methods on distance cycled, their trips are ‘worth less’ on average than trips by white males who tend to cycle further to work. While inclusion of health and carbon benefits in appraisal helps redress the balance in transport planning away from motorists' travel time, without incorporating an equity focus this may risk prioritising access to cycling for already privileged demographic groups.

## Funding statement

The Propensity to Cycle Tool project has been funded by the UK Department for Transport. Dr Woodcock and Dr Goodman have received funding from the European Research Council (ERC) under the Horizon 2020 research and innovation programme (grant agreement No 817754). This material reflects only the author's views and the Commission is not liable for any use that may be made of the information contained therein.

Dr Woodcock and Dr Goodman were also supported by the METAHIT Project (MRC Methodology Panel) (MR/P02663X/1).

## CRediT authorship contribution statement

**James Woodcock:** Conceptualization, Methodology, Writing – original draft, Supervision, Project administration, Funding acquisition. **Rachel Aldred:** Conceptualization, Methodology, Writing – review & editing, Funding acquisition. **Robin Lovelace:** Conceptualization, Methodology, Software, Writing – review & editing, Funding acquisition. **Tessa Strain:** Methodology, Writing – review & editing. **Anna Goodman:** Conceptualization, Methodology, Formal analysis, Writing – review & editing, Funding acquisition.

## Declaration of competing interest

None.

## Figures and Tables

**Fig. A1:**
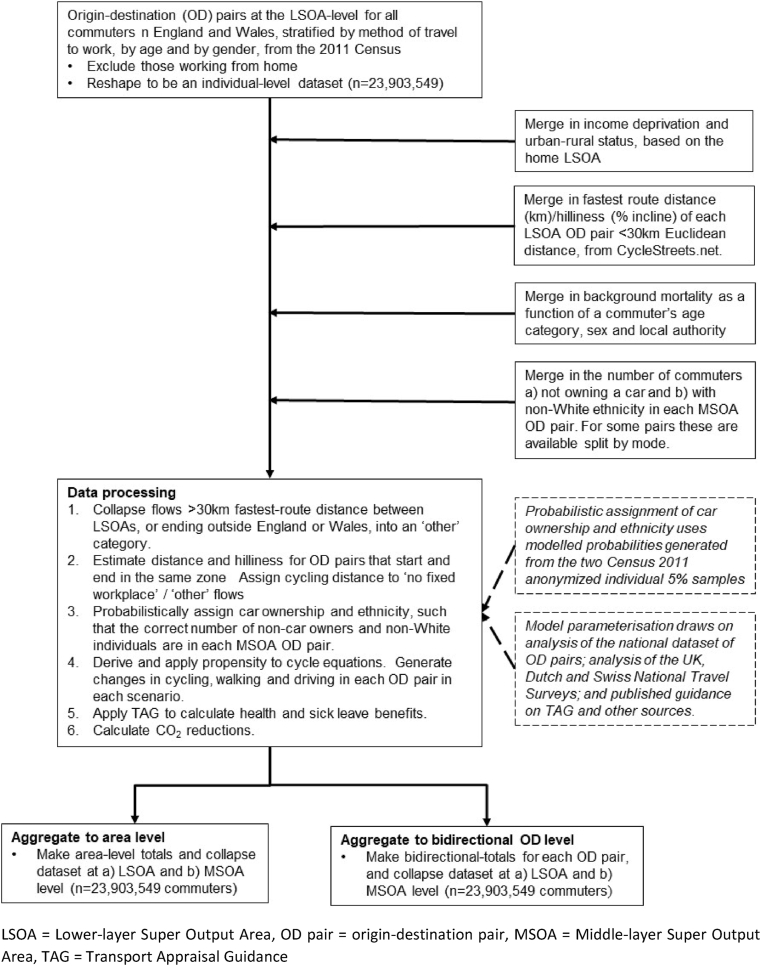
Flow diagram illustrating the input data and processing steps used to create the synthetic population of commuters from Census 2011) data, and then process it to generate PCT scenarios LSOA = Lower-layer Super Output Area, OD pair = origin-destination pair, MSOA = Middle-layer Super Output Area, TAG = Transport Appraisal Guidance

**Table A1:** Input or assumed speeds for uphill movement, used to develop decay function

Incline (%)	Speed (km/hr) based on data	Speed (km/hr) based on our decay function	Comments on data source
0	20	20	In 2015, the average moving speed of rides designated as commutes on Strava was 23.7 km/h^10^ but these are likely to be those going faster, with better bikes, over longer distances than for the typical commute. Strava data from cities outside the UK also gave average speeds of 20–25 km/h^11^ but the same biases likely apply. We took 20 km/h to be conservative, and this made it easier for us to match the observed NTS data.
0.75	16	16.3	0.75% is the average 2-way gradient for Cambridge. Average total journey speed when cycling for transport in Cambridge has been reported to be 16.1 km/h (Costa et al., 2015). 16 km/h is approximately the **uphill moving** speed one needs to assume to get this overall journey average if a) downhill speed is 20 km/h and b) 15% of the journey spent stationary (slightly lower than the assumed national average of 20%).
2.8	12.6	12.9	A study of 8 sedentary women averaged a speed of 12.6 km/h on a 3% short gradient (Sperlich et al., 2012). As these were sedentary women, we expect this slightly to underestimate the average commuter on this gradient.
5.0	9.9	10.4	A study of 8 sedentary women averaged a speed of 9.9 km/h on a 5% short gradient (Sperlich et al., 2012). As these were sedentary women, we expect this slightly to underestimate the average commuter on this gradient.
7.0	8	8.6	The lowest possible speed for a bike is in the range of 7.2 km/h^12^ but given many cycle up slopes of 10–15%, we expect the speed at 7% gradient to be higher than this minimum and set it as 8 km/h.

^10^https://bikmo.com/magazine/results-are-in-strava-reveals-average-british-cycle-commute-length/.

^11^https://www.vox.com/2015/10/8/9480951/bike-commute-data-strava.

^12^https://www.cyclist.co.uk/in-depth/682/how-steep-is-too-steep-when-cycling-uphill.

## Appendix 1 additional methods information

### Additional information on calculation of health benefits by hilliness

We sought to estimate the marginal MET (mMET) rate, and the associated average speed, involved in cycling on routes of different average gradient. In doing this, we were attempting to generate a plausible distribution of mMET values by hilliness, while not making large changes to the population average values for mMETs and speeds that are recommended by HEAT and TAG, and that the PCT had been using so far. In other words, we were seeking to refine the PCT approach to be more sensitive to differential effects across areas according to their hilliness, while retaining broadly similar overall estimates of health impact. It is for this reason that some of our assumptions were made with a view to back-fitting the output values to ultimately be consistent with the TAG and HEAT assumptions previously used in PCT.

Below we outline our methods for doing this, and the associated assumptions. These are also captured in the spreadsheet posted on GitHub at https://github.com/npct/pct-inputs/blob/master/02_intermediate/03_hilliness_calculations/EngWales_mmetspeed_hilliness.xlsx.

### Target range of hilliness values

In the 2011 Census, 99.9% of all commute routes had an average gradient of ≤7%. We therefore focused on estimating mMETs in this range, applying the 7% incline values to the small proportion of commuters who report travelling on steeper slopes.

### Power required in cycling

We used the equation from di Prampero et al. (Prampero et al., 1979) that calculates the power (in Watts) required by a cyclist to move. The equation can be broken up into three parts that we have termed road resistance, wind resistance, and gravity. These are then summed.

1. Road resistance = CoefficientofRollingResistance * Weight * GroundSpeed
2. Wind resistance = CoefficientofAirResistance * BodySurfaceArea *
(BarometricPressure/AirTemp) * AirVelocity^2^ * GroundSpeed
3. Gravity = Gravity *Weight * SineofAngleofIncline * GroundSpeed

Power (watts) = Road resistance + Wind Resistance + Gravity.

### Speed assumptions

#### Uphill moving speed by gradient

We used the data points in Table A1 to develop a decay function for uphill moving speed with incline. We fit this decay function using linear regression, with speed as the outcome and the square root of incline as the predictor.

From these data points we used the following formula for our assumption of uphill moving speed as average gradient (as a percentage) increased:

Speed = 20–4.3 * (gradient ^ 0.5)

As shown in the third column of Table A1, this provided a relatively good fit to our input data.

#### Stationary time in each journey

We further assumed there was a proportion of each journey spent stationary (e.g. waiting for traffic lights and stuck in traffic) and so speed when moving would be different from total journey speed.

Total journey speed = speed when moving * proportion of time spent moving

In practice this is a simplification of the reality in which a cyclist spends some time stationary, some time travelling slowly e.g. because of traffic, and some time travelling at a steady-state speed.

We assumed the MET value of stationary time was equal to the MET value for steady-speed cycling. We made this simplifying assumption to balance out the low energy requirements of waiting stationary at a traffic light (plausibly around 2–3 MET) versus the higher energy requirements involved in the start-stop nature of cycling when interacting with other road users.

Accelerating requires more energy than holding a constant speed. However, we do not have the data to model in detail acceleration and waiting. Thus we assumed that on average the lost time was at the average MMET rate as for the whole journey, rather than at a resting rate. This also provided a much better fit with observed objective data e.g. Costa than assuming the time was spent resting.

We assumed 20% of total journey time could be spent stationary, based on numbers discussed in various London cycling blogs of 10–30%.^10^ We selected 20% within this range as a value that gave a fairly close match between average speed in this new method and the average previously used of 14 km/h.

It is likely that better cycling infrastructure e.g. under a Go Dutch scenario would reduce this time but we lack data to include this in the quantitative model.

### Other assumptions

#### Ground Resistance Coefficient

We assumed this to be 0.007 because 0.005 is typical for standard road surface with clincher tyres^11^; we assumed a worse road surface and poorly maintained tyres on commuter cyclists.

#### Weight of rider

We assumed this to be 76.9 kg as an average between the English average male (83.6 kg) and female (70.2 kg).^12^

#### Weight of bike and bags

We assumed this to be 16 kg as a good commuter bike can weigh approximately 11–12 kg^13^ and we added on 3–4 kg for a bag and other bike accessories.

#### Wind Resistance Coefficient

We assumed this to be 0.5, which indicates no head or tailwind.^14^

#### Frontal Area

We assumed this to be 0.8 m^2^ as 0.63 is typical for “tops” position^15^; we estimate a bit higher for upright commuters with non-aerodynamic bags and clothing. This is fractionally higher than the value given in theclimbingcyclist blog^16^ (0.6 m^2^) but commuter cyclists are more likely to sit very upright even when on “tops” and so this rounding up is probably warranted.

#### Air density and gravity

These were set at 1.225 kg/m^2^ (roughly sea level and 15° temperature) and 9.8 m/s.^2^ Air density is pressure/air temperature.

#### Efficiency

Not all power generated will be transferred to the bike. A well-maintained bike is thought to be about 95% efficient.^17^ The lower end of the range (where we expect commuters to be) is around 93%^18^; this was the value we used.

### Conversion from Watts to mMETs

We used the equation from the Hawley and Noakes (1992) paper showing a very high correlation between Max power output (Wmax) and VO_2_ max to convert Watts to L/min of O_2_ (Hawley and Noakes 1992).

VO_2_ max = 0.01141 x Wmax +0.435

We then converted from L/min to kcal/min by multiplying by 5.^19^ This was then divided by bodyweight and multiplied the time spent moving in hours.

Marginal METs were calculated by subtracting 1 MET.

#### Calculating average METs and speeds for two-way trips

The PCT is based on average gradients, i.e. a gradient of 1.5% means an average uphill gradient of 1.5% in one direction, and average downhill gradient of 1.5% in the other direction. We assumed that energy expenditure and speed when going downhill was equal to energy expenditure and speed when travelling on the flat.

#### Estimating energy expenditure for walking and e-biking

We assumed the relative effort of walking on a hill was directly proportional to the relative effort of cycling on a hill. We therefore multiplied all our cycling mMET values by 0.663, the ratio that gave an overall average walking mMET value of 3.6. This is the value that has been reported in the literature, and that we have been using so far in PCT (Costa et al., 2015).

We assumed that having an e-bike halved the additional effort required when going uphill, which is in line with our previous observation that the deterrent effect of hills for e-bike-owners was around half the size as non-e-bike owners (Lovelace et al., 2017). We further assumed that cycling on the flat was 1.8 mMET less effort on an e-bike than on a bicycle. Together this approximately generated the average e-biking mMET of 3.5 that has been reported in the literature, and that we have been using so far in PCT (Sperlich et al., 2012).

#### Estimating speeds for walking and e-biking

We assumed the relative speed penalty of walking on a hill was directly proportional to the relative speed penalty of cycling on a hill. Thus far in PCT we have been assuming cycling speeds of 14 km/h and walking speeds of 4.8 km/h, based on HEAT guidance (Kahlmeier et al., 2014, page 16).^20^ We multiplied all our newly-calculated cycling speeds values by 4.8/14 = 0.34 to give an updated estimate of walking speed by gradient.

For e-biking, we thought it plausible that the relative speed penalty of travelling on a hill would be smaller than for a traditional bike. We had previously been assuming that e-biking speed was 1.17 times faster than cycling speed. This was based on the Dutch NTS 2013–2016, in which mean cycling speed was 15.0 km/h for bicycle commute trips and 17.5 km/h for e-bike commute trips (17.5/15.0 = 1.17). We have also estimated that the average route gradient in the Netherlands is 0.78%. We therefore applied this ratio of 1.17 to routes with an average gradient of 0.75%. For lower and higher gradients, we scaled this such that the hilliness effect was half that observed for cycling.

## Footnotes

2 We define propensity as the modelled likelihood of cycling a trip either currently or based on some scenario. Potential is a normative measure of what could be achieved, and we use propensity estimates from e.g. the Netherlands to estimate. For example women currently have lower cycling propensity than men in England but applying Dutch propensity to their current trips they have greater cycling potential.

3 See also Pajarito and Gould, 2018 and Raffler et al., (2019) for alternative approaches to modelling these kinds of trip characteristics in relation to cycling potential.

4 https://npct.github.io/pct-shiny/regions_www/www/static/03d_other_reports/2020-pct-impact-report.pdf.

5 While Census origin-destination data is generally open access, we had to request safeguarded data (available to researchers) to create the PCT.

6 The target refers to trip stages rather than trips, but currently in England, these are similar for cycling as a mode.

7 https://www.pct.bike/tabs/manual.html.

8 All trip characteristics (distance, hilliness, walking and driving mode shares) are directly taken from the data for all demographic groups.

9 Currently the carbon prices used by the Department for Transport results in only a small economic value relative to health or time savings but new higher values are being introduced in sensitivity analysis, and even higher prices may be appropriate for authorities with more ambitious targets. https://www.gov.uk/government/publications/tag-forthcoming-changes-to-carbon-values.

Appendix A Supplementary data to this article can be found online at https://doi.org/10.1016/j.jth.2021.101066.
