# Supplementary material for: Health, environmental and distributional impacts of cycling uptake: The model underlying the Propensity to Cycle tool for England and Wales
Source: J Transp Health. 2021 Sep;22:101066. doi: 10.1016/j.jth.2021.101066 (PMC8463831; doi:10.1016/j.jth.2021.101066)
Supplement: Multimedia component 1 [file mmc1.docx]

### Appendix 2: supplementary results

Deaths averted per year, in total and disaggregated by group

|  |  | No. commuters | Baseline (changes relative to no cycling) | Scenarios (changes relative to baseline) |  |  |  |  |
| --- | --- | --- | --- | --- | --- | --- | --- | --- |
|  |  |  |  | Near market | Equality | Gender Equality | Go Dutch | E-bikes |
| Whole sample |  | 23,903,549 | 198 | 211 | 217 | 74 | 939 | 1062 |
| Sex | Male | 12,467,760 | 167 | 180 | 137 | 0 | 584 | 684 |
|  | Female | 11,435,789 | 31 | 31 | 79 | 74 | 355 | 378 |
| Age | 16 to 24 | 3,237,168 | 3 | 3 | 3 | 0.4 | 12 | 13 |
|  | 25 to 34 | 5,538,697 | 16 | 15 | 11 | 3 | 44 | 50 |
|  | 35 to 49 | 8,650,594 | 62 | 66 | 47 | 17 | 199 | 228 |
|  | 50 to 64 | 5,804,849 | 86 | 97 | 110 | 40 | 481 | 548 |
|  | 65+ | 672,241 | 30 | 30 | 46 | 14 | 203 | 224 |
| Ethnicity | White | 21,050,896 | 188 | 199 | 195 | 69 | 843 | 962 |
|  | Non-white | 2,852,653 | 11 | 12 | 22 | 5 | 96 | 100 |
| Household car | 1 or more cars | 20,703,404 | 154 | 181 | 197 | 67 | 856 | 985 |
|  | No car | 3,200,146 | 44 | 30 | 20 | 7 | 83 | 77 |
| Income | Fifth 1 (poorest) | 4,076,504 | 29 | 31 | 33 | 9 | 154 | 159 |
| deprivation | Fifth 2 | 4,872,476 | 38 | 40 | 41 | 13 | 180 | 195 |
|  | Fifth 3 | 5,060,155 | 43 | 44 | 45 | 16 | 195 | 221 |
|  | Fifth 4 | 4,996,073 | 44 | 47 | 48 | 18 | 205 | 241 |
|  | Fifth 5 (richest) | 4,898,341 | 44 | 50 | 49 | 19 | 205 | 246 |
| Urban/rural | Rural | 4,103,067 | 33 | 32 | 38 | 15 | 152 | 202 |
|  | Urban | 19,800,482 | 165 | 179 | 178 | 59 | 787 | 860 |

Years of Life Lost (YLLs) averted per year, in total and disaggregated by group

|  |  | No. commuters | Baseline (changes relative to no cycling) | Scenarios (changes relative to baseline) |  |  |  |  |
| --- | --- | --- | --- | --- | --- | --- | --- | --- |
|  |  |  |  | Near market | Equality | Gender Equality | Go Dutch | E-bikes |
| Whole sample |  | 23,903,549 | 5,454 | 5,830 | 5,624 | 1,922 | 24,273 | 27,520 |
| Sex | Male | 12,467,760 | 4,680 | 5,020 | 3,579 | 0 | 15,146 | 17,755 |
|  | Female | 11,435,789 | 774 | 810 | 2,045 | 1,922 | 9,128 | 9,765 |
| Age | 16 to 24 | 3,237,168 | 149 | 136 | 120 | 17 | 531 | 554 |
|  | 25 to 34 | 5,538,697 | 621 | 584 | 426 | 124 | 1,754 | 1,974 |
|  | 35 to 49 | 8,650,594 | 2,063 | 2,192 | 1,565 | 546 | 6,595 | 7,561 |
|  | 50 to 64 | 5,804,849 | 2,197 | 2,465 | 2,805 | 1,017 | 12,241 | 13,940 |
|  | 65+ | 672,241 | 424 | 452 | 708 | 218 | 3,153 | 3,491 |
| Ethnicity | White | 21,050,896 | 5,134 | 5,472 | 5,011 | 1,778 | 21,600 | 24,717 |
|  | Non-white | 2,852,653 | 321 | 358 | 613 | 144 | 2,674 | 2,804 |
| Household | 1 or more cars | 20,703,404 | 4,236 | 4,977 | 5,101 | 1,737 | 22,095 | 25,489 |
| car | No car | 3,200,146 | 1,218 | 853 | 523 | 185 | 2,179 | 2,031 |
| Income | Fifth 1 (poorest) | 4,076,504 | 812 | 888 | 896 | 238 | 4,111 | 4,242 |
| deprivation | Fifth 2 | 4,872,476 | 1,070 | 1,123 | 1,081 | 342 | 4,735 | 5,163 |
|  | Fifth 3 | 5,060,155 | 1,189 | 1,223 | 1,183 | 411 | 5,058 | 5,745 |
|  | Fifth 4 | 4,996,073 | 1,186 | 1,266 | 1,231 | 454 | 5,209 | 6,160 |
|  | Fifth 5 (richest) | 4,898,341 | 1,197 | 1,329 | 1,234 | 477 | 5,161 | 6,209 |
| Urban/rural | Rural | 4,103,067 | 843 | 839 | 963 | 377 | 3,798 | 5,072 |
|  | Urban | 19,800,482 | 4,611 | 4,991 | 4,661 | 1,545 | 20,475 | 22,448 |

Reduction in person-years of sickness absenteeism per year, in total and disaggregated by group

|  |  | No. commuters | Baseline (changes relative to no cycling) | Scenarios (changes relative to baseline) |  |  |  |  |
| --- | --- | --- | --- | --- | --- | --- | --- | --- |
|  |  |  |  | Near market | Equality | Gender Equality | Go Dutch | E-bikes |
| Whole sample |  | 23,903,549 | 1,878 | 1,981 | 2,107 | 1,068 | 9,910 | 11,869 |
| Sex | Male | 12,467,760 | 1,430 | 1,505 | 991 | 0 | 4,589 | 5,739 |
|  | Female | 11,435,789 | 448 | 476 | 1,115 | 1,068 | 5,321 | 6,130 |
| Age | 16 to 24 | 3,237,168 | 133 | 111 | 152 | 80 | 720 | 805 |
|  | 25 to 34 | 5,538,697 | 432 | 410 | 391 | 220 | 1,778 | 2,123 |
|  | 35 to 49 | 8,650,594 | 716 | 781 | 711 | 381 | 3,329 | 4,025 |
|  | 50 to 64 | 5,804,849 | 564 | 643 | 794 | 364 | 3,798 | 4,580 |
|  | 65+ | 672,241 | 33 | 36 | 59 | 22 | 285 | 336 |
| Ethnicity | White | 21,050,896 | 1,759 | 1,850 | 1,858 | 969 | 8,732 | 10,553 |
|  | Non-white | 2,852,653 | 118 | 131 | 249 | 98 | 1,179 | 1,315 |
| Household car | 1 or more cars | 20,703,404 | 1,429 | 1,672 | 1,891 | 947 | 8,944 | 10,895 |
|  | No car | 3,200,146 | 448 | 309 | 216 | 121 | 967 | 973 |
| Income | Fifth 1 (poorest) | 4,076,504 | 320 | 346 | 387 | 158 | 1,905 | 2,088 |
| Deprivation | Fifth 2 | 4,872,476 | 387 | 397 | 423 | 205 | 2,003 | 2,320 |
|  | Fifth 3 | 5,060,155 | 400 | 408 | 435 | 227 | 2,025 | 2,434 |
|  | Fifth 4 | 4,996,073 | 388 | 413 | 438 | 235 | 2,025 | 2,539 |
|  | Fifth 5 (richest) | 4,898,341 | 383 | 417 | 425 | 242 | 1,953 | 2,487 |
| Urban/rural | Rural | 4,103,067 | 244 | 252 | 310 | 164 | 1,365 | 1,974 |
|  | Urban | 19,800,482 | 1,634 | 1,730 | 1,797 | 904 | 8,546 | 9,895 |

Millions of pounds of health economic benefit (YLL + sickness absence) per year, in total and disaggregated by group

|  |  | No. commuters | Baseline (changes relative to no cycling) | Scenarios (changes relative to baseline) |  |  |  |  |
| --- | --- | --- | --- | --- | --- | --- | --- | --- |
|  |  |  |  | Near market | Equality | Gender Equality | Go Dutch | E-bikes |
| Whole sample |  | 23,903,549 | 416 | 442 | 436 | 167 | 1,923 | 2,211 |
| Sex | Male | 12,467,760 | 347 | 369 | 259 | 0 | 1,116 | 1,326 |
|  | Female | 11,435,789 | 69 | 73 | 177 | 167 | 807 | 885 |
| Age | 16 to 24 | 3,237,168 | 16 | 14 | 15 | 5 | 68 | 74 |
|  | 25 to 34 | 5,538,697 | 60 | 56 | 46 | 19 | 197 | 227 |
|  | 35 to 49 | 8,650,594 | 157 | 168 | 128 | 52 | 555 | 646 |
|  | 50 to 64 | 5,804,849 | 157 | 176 | 204 | 78 | 906 | 1,044 |
|  | 65+ | 672,241 | 26 | 28 | 44 | 14 | 197 | 220 |
| Ethnicity | White | 21,050,896 | 391 | 414 | 386 | 153 | 1,701 | 1,974 |
|  | Non-white | 2,852,653 | 25 | 28 | 50 | 14 | 222 | 237 |
| Household | 1 or more cars | 20,703,404 | 321 | 375 | 394 | 150 | 1,742 | 2,038 |
| car | No car | 3,200,146 | 95 | 67 | 43 | 18 | 181 | 173 |
| Income | Fifth 1 (poorest) | 4,076,504 | 64 | 69 | 72 | 22 | 338 | 354 |
| deprivation | Fifth 2 | 4,872,476 | 83 | 86 | 85 | 31 | 381 | 422 |
|  | Fifth 3 | 5,060,155 | 90 | 93 | 91 | 36 | 399 | 460 |
|  | Fifth 4 | 4,996,073 | 89 | 95 | 94 | 38 | 406 | 487 |
|  | Fifth 5 (richest) | 4,898,341 | 89 | 99 | 93 | 40 | 400 | 488 |
| Urban/rural | Rural | 4,103,067 | 61 | 61 | 71 | 30 | 288 | 392 |
|  | Urban | 19,800,482 | 355 | 381 | 365 | 137 | 1,635 | 1,819 |

Reduction in thousands of tonnes of transport CO2 equivalent per year, in total and disaggregated by group

|  |  | No. commuters | Baseline (changes relative to no cycling) | Scenarios (changes relative to baseline) |  |  |  |  |
| --- | --- | --- | --- | --- | --- | --- | --- | --- |
|  |  |  |  | Near market | Equality | Gender Equality | Go Dutch | E-bikes |
| Whole sample |  | 23,903,549 | 104 | 115 | 112 | 43 | 496 | 859 |
| Sex | Male | 12,467,760 | 86 | 95 | 63 | 0 | 271 | 480 |
|  | Female | 11,435,789 | 18 | 19 | 49 | 43 | 225 | 379 |
| Age | 16 to 24 | 3,237,168 | 13 | 9 | 10 | 4 | 47 | 80 |
|  | 25 to 34 | 5,538,697 | 25 | 25 | 23 | 9 | 101 | 176 |
|  | 35 to 49 | 8,650,594 | 43 | 53 | 43 | 17 | 189 | 330 |
|  | 50 to 64 | 5,804,849 | 21 | 26 | 32 | 13 | 143 | 247 |
|  | 65+ | 672,241 | 2 | 2 | 3 | 1 | 16 | 27 |
| Ethnicity | White | 21,050,896 | 98 | 109 | 101 | 41 | 446 | 778 |
|  | Non-white | 2,852,653 | 6 | 6 | 11 | 3 | 50 | 80 |
| Household | 1 or more cars | 20,703,404 | 83 | 108 | 109 | 42 | 481 | 833 |
| car | No car | 3,200,146 | 22 | 7 | 3 | 1 | 15 | 25 |
| Income | Fifth 1 (poorest) | 4,076,504 | 15 | 16 | 16 | 5 | 76 | 124 |
| deprivation | Fifth 2 | 4,872,476 | 19 | 21 | 21 | 7 | 94 | 158 |
|  | Fifth 3 | 5,060,155 | 22 | 24 | 24 | 9 | 105 | 182 |
|  | Fifth 4 | 4,996,073 | 24 | 26 | 26 | 11 | 111 | 197 |
|  | Fifth 5 (richest) | 4,898,341 | 24 | 28 | 26 | 11 | 110 | 198 |
| Urban/rural | Rural | 4,103,067 | 18 | 19 | 22 | 10 | 86 | 170 |
|  | Urban | 19,800,482 | 86 | 95 | 91 | 34 | 410 | 689 |
